# Phosphoinositides and the Fate of *Legionella* in Phagocytes

**DOI:** 10.3389/fimmu.2020.00025

**Published:** 2020-01-30

**Authors:** A. Leoni Swart, Hubert Hilbi

**Affiliations:** Faculty of Medicine, Institute of Medical Microbiology, University of Zürich, Zurich, Switzerland

**Keywords:** *Dictyostelium discoideum*, effector protein, endoplasmic reticulum, host-pathogen interaction, macrophage, pathogen vacuole, type IV secretion, vesicle trafficking

## Abstract

*Legionella pneumophila* is the causative agent of a severe pneumonia called Legionnaires' disease. The environmental bacterium replicates in free-living amoebae as well as in lung macrophages in a distinct compartment, the *Legionella*-containing vacuole (LCV). The LCV communicates with a number of cellular vesicle trafficking pathways and is formed by a plethora of secreted bacterial effector proteins, which target host cell proteins and lipids. Phosphoinositide (PI) lipids are pivotal determinants of organelle identity, membrane dynamics and vesicle trafficking. Accordingly, eukaryotic cells tightly regulate the production, turnover, interconversion, and localization of PI lipids. *L. pneumophila* modulates the PI pattern in infected cells for its own benefit by (i) recruiting PI-decorated vesicles, (ii) producing effectors acting as PI interactors, phosphatases, kinases or phospholipases, and (iii) subverting host PI metabolizing enzymes. The PI conversion from PtdIns(3)*P* to PtdIns(4)*P* represents a decisive step during LCV maturation. In this review, we summarize recent progress on elucidating the strategies, by which *L. pneumophila* subverts host PI lipids to promote LCV formation and intracellular replication.

## *Legionella Pneumophila*—An Amoebae-Resistant Environmental Bacterium

*Legionella* spp. are obligate aerobic, Gram-negative bacteria, which are ubiquitously found in technical and natural water systems, where they colonize different niches (1, 2). The facultative intracellular bacteria replicate in planktonic form as well as in biofilms (3–5), and they infect environmental predators such as nematodes (6–9) and protozoa (10–12). Complex, ecologically relevant interactions take place in the aquatic niches inhabited by *Legionella* spp.; e.g., nematode larvae rupture *Legionella*-infected amoebae and thus are exposed to a highly virulent form of the bacterial pathogen (9).

Upon inhalation of contaminated water droplets, *Legionella* bacteria reach the lung, where they replicate in and destroy alveolar macrophages, thus causing a potentially fatal pneumonia termed Legionnaires' disease (2). The clinically most relevant and best studied species is *Legionella pneumophila*; yet, *Legionella longbeachae* is prevalent in some parts of the world, too (13). The spread of *Legionella* spp. predominantly occurs through environmental sources; however, a probable person-to-person transmission of *L. pneumophila*, resulting in the death of the two people involved, was recently reported (14).

*Legionella pneumophila* replicates intracellularly in amoebae and macrophages by exploiting evolutionarily conserved pathways (15, 16). The pathogen forms a unique, degradation-resistant compartment, the *Legionella*-containing vacuole (LCV), wherein which bacterial replication takes place. The LCV does neither acidify nor fuse with lysosomes, but communicates with several vesicle trafficking pathways including the endosomal, secretory, and retrograde routes (17–21). At later steps of pathogen vacuole maturation, the LCV tightly and continuously associates with the endoplasmic reticulum (ER). Small GTPases of the Arf (22, 23), Rab (24, 25), Ran (26), and Rap (27) families regulate LCV formation and intracellular replication of *L. pneumophila*. Moreover, large GTPases implicated in eukaryotic membrane fusion and fission play a role in *L. pneumophila* infection. Atlastin3 (Atl3/Sey1), an ER tubule-resident large GTPase that catalyzes homotypic ER fusions, promotes ER remodeling around LCVs, pathogen vacuole expansion and intracellular bacterial replication (28). Dynamin1-like GTPase (Dnm1l), a mitochondrial large GTPase, mediates *L. pneumophila*-induced mitochondrial fragmentation and inhibition of host cell respiration (29).

LCV formation requires the Icm/Dot (intracellular multiplication/defective organelle trafficking) type IVB secretion system (T4SS), which is conserved among *Legionella* spp., and in the case of *L. pneumophila* translocates more than 300 different “effector” proteins into host cells (30, 31). In eukaryotic cells, the effector proteins subvert essential process such as signal transduction, cytoskeleton dynamics and membrane trafficking (17, 32–37). Distinct effector proteins have been shown to target the small GTPases Arf1 (22), Rab1 (38–41) or Ran (26, 42), the retromer coat complex (43–46), the vacuolar H^+^-ATPase (47), the autophagy machinery (48–50), or phosphoinositide (PI) lipids (35, 51, 52). Here, we focus on how *L. pneumophila* subverts host PI lipids to promote LCV formation and intracellular replication.

## Phosphoinositide lipids—Regulators of Organelle Identity and Membrane Dynamics

**Figure 1:**
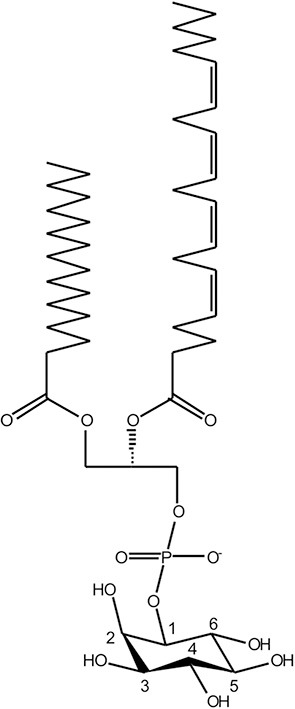
Chemical structure of phosphoinositide lipids. The core moiety of phosphoinositide (PI) lipids is phosphatidylinositol (PtdIns), comprising diacylglycerol (DAG), and D-*myo*-inositol 1-phosphate. The inositol head group is reversibly phosphorylated by organelle-specific PI kinases and PI phosphatases at the positions 3, 4, and/or 5, giving rise to seven different mono- or poly-phosphorylated derivatives.

Phosphoinositides are minor constituents of eukaryotic membranes (<10% of all phospholipids), but this low abundance class of lipids exert pivotal functions for cellular organelle identity, membrane dynamics and vesicle trafficking (53–56). Accordingly, the production, turnover, interconversion, and subcellular localization of PI lipids are tightly regulated by eukaryotic cells. The core compound of PI lipids is phosphatidylinositol (PtdIns), comprising a diacylglycerol (DAG) moiety and a D-*myo*-inositol 1-phosphate head group facing the cytoplasmic side of membranes (Figure 1). PtdIns can be reversibly phosphorylated at the positions 3, 4, and/or 5 of the inositol ring, giving rise to seven different mono- or poly-phosphorylated derivatives (53–56). These reactions are catalyzed by organelle-specific PI metabolizing enzymes (PI kinases and PI phosphatases), the activity of which controls compartmentalization and vesicle trafficking within the cell (57, 58).

PI lipids, jointly with small GTPases in their active GTP-bound form, recruit peripheral membrane proteins harboring distinct PI-binding motifs, such as the PH, PX, FYVE, ENTH/ANTH, or FERM domains (59). Hence, lipid-protein co-incidence detection, along with specific adaptor proteins, determines organelle identity and vesicle trafficking routes in eukaryotic cells (54, 60). PI-metabolizing enzymes are usually recruited to the cytoplasmic side of cellular membranes by small GTPases; e.g., the endosomal small GTPase Rab5 recruits and activates the class III phosphatidylinositol 3-kinase (PI3K) to produce PtdIns(3)*P* from PtdIns (61). The small GTPases themselves are localized and activated by specific guanine nucleotide exchange factors (GEFs), which concomitantly displace the guanine nucleotide dissociation inhibitor (GDI) protein from the small GTPase, thus allowing the membrane association of the GTPase. To switch off the signal, the inactivation of small GTPases is catalyzed by specific GTPase activating proteins (GAPs) (61).

**Figure 2:**
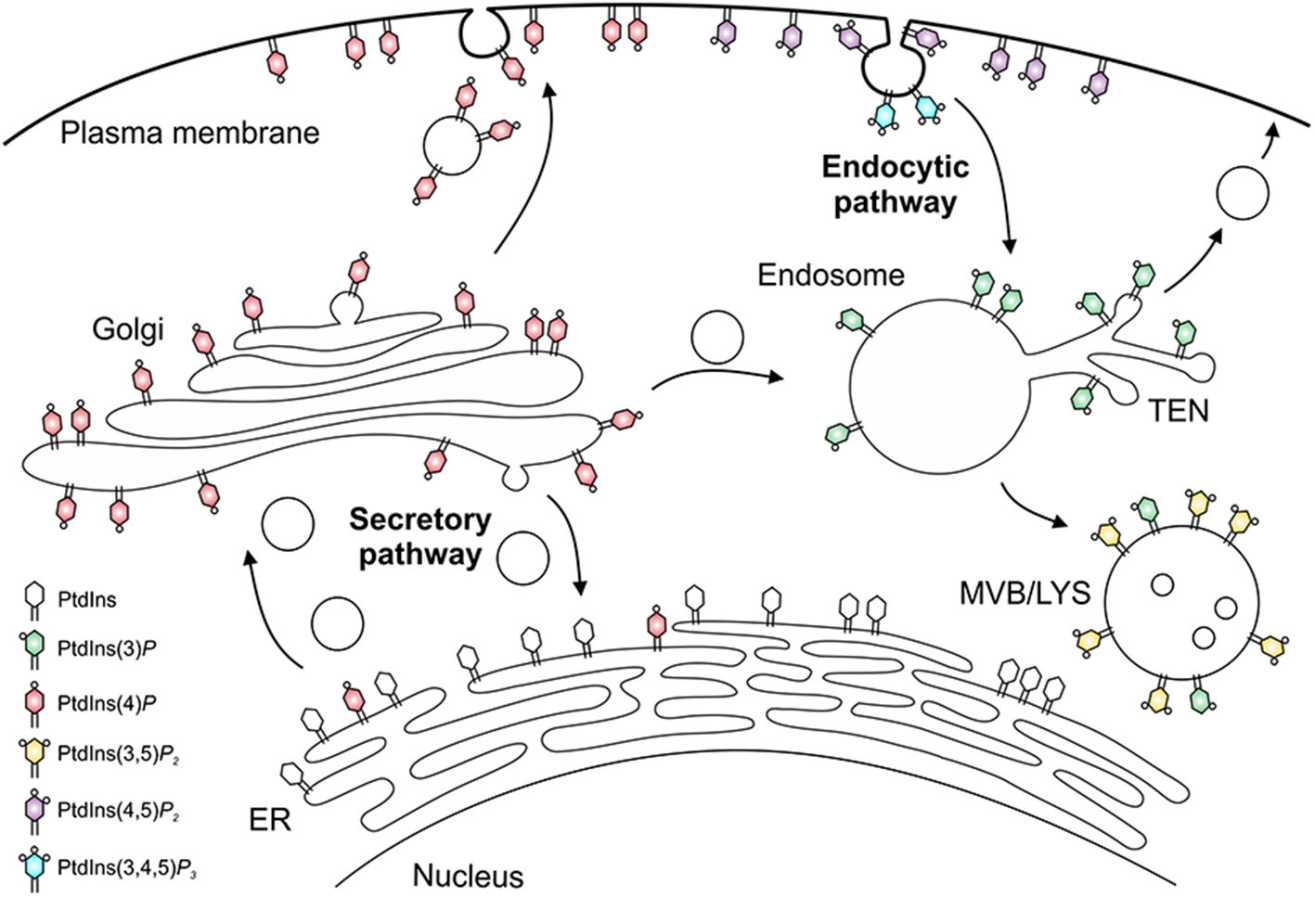
Subcellular distribution of phosphoinositides. The subcellular distribution of phosphoinositide lipids is primarily arranged around the cellular dichotomy of the secretory and endocytic vesicle trafficking pathways. In the secretory pathway, PtdIns(4)*P* is synthesized in the endoplasmic reticulum (ER) and Golgi apparatus, localizes to secretory vesicles, and finally accumulates at the plasma membrane, where it is converted to PtdIns(4,5)*P*_2_ and, transiently, to PtdIns(3,4,5)*P*_3_. In the endocytic pathway, PtdIns(3)*P* decorates early endosomes and the tubular endosomal network (TEN), and is converted to PtdIns(3,5)*P*_2_ on multivesicular bodies (MVB), late endosomes and lysosomes (LYS).

The different PIs preferentially localize to distinct subcellular compartments and pathways [(53, 54, 62); Figure 2]. Accordingly, PtdIns(4)*P* and in particular PtdIns(4,5)*P*_2_ are enriched at the plasma membrane, where PtdIns(3,4,5)*P*_3_ and PtdIns(3,4)*P*_2_ transiently accumulate upon signal transduction events and during phagocytosis. PtdIns(3)*P* is the “signpost” PI lipid of the endocytic pathway, and is enriched on phagosomes and early endosomes, as well as on autophagosomes and multivesicular bodies, which like late endosomes and lysosomes are also decorated with PtdIns(3,5)*P*_2_. PtdIns(4)*P* is the hallmark PI lipid of the secretory pathway and predominantly localizes to the Golgi apparatus and secretory vesicles (53, 54, 56, 62). This PI lipid is formed from PtdIns on the ER and together with PtdIns(3)*P* also regulates phagosome-lysosome fusion (63).

On certain compartments and along some vesicle trafficking pathways, distinct PIs are functionally coupled, i.e., the product of a given PI-metabolizing enzyme is the substrate of a subsequent modification. This occurs, e.g., in the endocytic pathway, where PtdIns(3)*P* is phosphorylated to yield PtdIns(3,5)*P*_2_, as well as in the secretory pathway, where PtdIns(4)*P* serves as the precursor of PtdIns(4,5)*P*_2_ at the plasma membrane. In turn, PtdIns(4,5)*P*_2_ is phosphorylated by class I PI3K to transiently yield PtdIns(3,4,5)*P*_3_ during phagocytosis.

## Eukaryotic PI Kinases Implicated in Uptake and Endocytosis of *L. Pneumophila*

PtdIns(3,4,5)*P*_3_ and PtdIns(3)*P* are produced by class I or class III PI3Ks and are major regulators of phagocytosis or the endocytic pathway, respectively. Using the haploid social soil amoeba *Dictyostelium discoideum*, genetic and pharmacological disruption of class I PI3Ks indicated that these kinases are largely dispensable for uptake of wild-type *L. pneumophila*, but required for uptake of an *icm*/*dot* mutant strain (51, 64). Moreover, using *D. discoideum* producing a fluorescent probe for PtdIns(3,4,5)*P*_3_, live-cell microscopy revealed that this PI lipid accumulated at bacterial entry sites and was cleared within approximately 40 s after uptake, regardless of whether the amoebae were infected with wild-type or *icm*/*dot* mutant *L. pneumophila*. In parallel, plasma membrane PtdIns(4,5)*P*_2_ disappeared from the uptake sites (65).

Similar to amoebae, the uptake of *L. pneumophila* wild-type, but not the *icm*/*dot* mutant strain by replication-permissive human U937 macrophage-like cells was not affected by the class I PI3K inhibitor wortmannin (66, 67). In contrast, wortmannin or LY294002 inhibited the uptake of wild-type as well as *icm*/*dot* mutant *L. pneumophila* by non-permissive murine J774A.1 macrophages (64, 66, 67). The Icm/Dot T4SS controls the uptake of *L. pneumophila* by phagocytes (68, 69); however, no effectors implicated in the process have been identified. These results suggest that during uptake of *L. pneumophila* class I PI3Ks are activated and the pathogen evades/inhibits downstream processes in an Icm/Dot-dependent manner to form the replication-permissive compartment.

*Dictyostelium discoideum* mutant strains were also used to examine the role of endosomal PI kinases, PI phosphatases and phospholipases for intracellular growth of *L. pneumophila*. Wild-type *L. pneumophila* replicated more efficiently in *D. discoideum* lacking two or five class I PI3Ks (51, 64) or in amoebae lacking PIKfyve (70), a PI 5-kinase, which is recruited through its FYVE domain to early endosomes, where it phosphorylates PtdIns(3)*P* to yield PtdIns(3,5)*P*_2_. While it is not clear how lower levels of PtdIns(3,4,5)*P*_3_ promote the intracellular replication of *L. pneumophila*, the reduction of PtdIns(3,5)*P*_2_ impairs the bactericidal endocytic pathway, which restricts bacterial killing and thus benefits the pathogen (70). The disruption of *D. discoideum* PTEN (phosphatase and tensin homolog), a PI phosphatase antagonizing PI3Ks, reduces the uptake of *L. pneumophila* but does not affect intracellular growth (64). Finally, the inhibition of *D. discoideum* PLC (Phospholipase C), a hydrolase cleaving PI(4,5)*P*_2_ to yield DAG and inositol 1,4,5-phosphate (IP_3_), also abolishes the uptake of *L. pneumophila*, but again has no effect on bacterial replication (64).

## Phosphoinositide Conversion on the *Legionella*-Containing Vacuole

**Figure 3:**
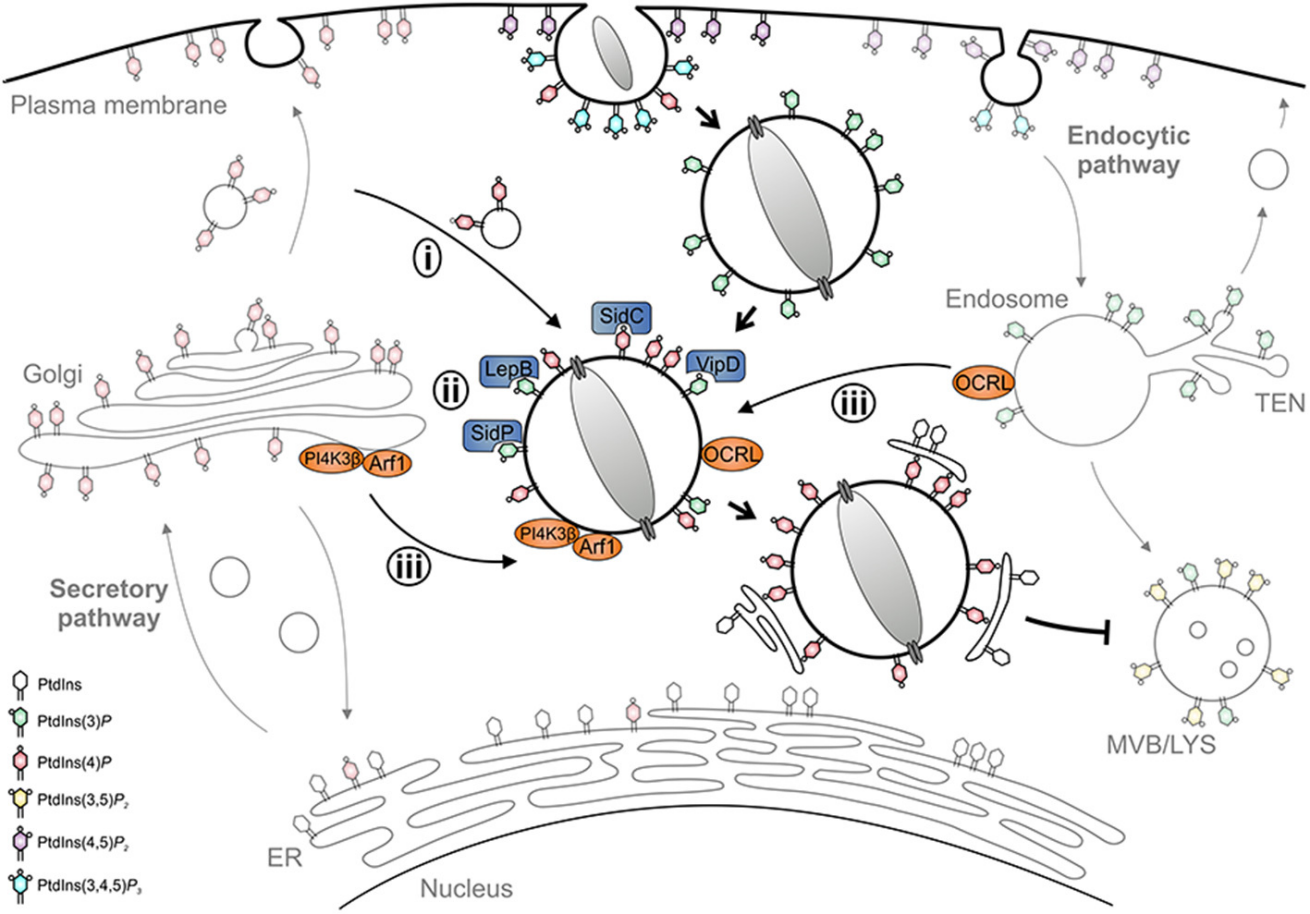
LCV formation and phosphoinositide conversion. The *Legionella*-containing vacuole (LCV) is a replication-permissive compartment disconnected from the bactericidal endocytic pathway and tightly associated with the ER. LCV formation is governed by a PI conversion from endosomal PtdIns(3)*P* to secretory PtdIns(4)*P*. *L. pneumophila* subverts the LCV PI pattern (i) by recruiting and selectively retaining PI-decorated vesicles, (ii) by producing effectors acting as PI interactors (SidC), kinases (LepB), phosphatases (SidP), or phospholipases (VipD), and (iii) by subverting host PI kinases (PI4KIIIβ) and phosphatases (OCRL).

PtdIns(3)*P* accumulates on LCVs within 1 min after uptake, regardless of whether the vacuole contains wild-type or *icm*/*dot* mutant *L. pneumophila* (71). However, while phagosomes containing *icm*/*dot* mutant bacteria remain decorated with PtdIns(3)*P*, more than 80% of wild-type LCVs gradually lose this PI within 2 h. Concomitantly, major membrane rearrangements take place with PtdIns(3)*P*-positive membranes being segregated from the LCV and compacted at the cell center. PtdIns(4)*P*, on the other hand, transiently localizes to early phagosomes harboring wild-type or *icm*/*dot* mutant *L. pneumophila*, but is cleared within minutes after uptake. During the following 2 h, PtdIns(4)*P* steadily accumulates only on wild-type LCVs, which for at least 8 h maintain a discrete PtdIns(4)*P* identity spatially separated from the calnexin-positive ER. PtdIns(4)*P* decorates the LCV for a prolonged time (18 h p. i. and beyond) up to when the bacteria exit from the pathogen vacuole and the infected cell (71). Taken together, within 2 h post-infection, the LCV undergoes a PI conversion, replacing the endosomal PtdIns(3)*P* with the secretory PtdIns(4)*P* (Figure 3). Importantly, the LCV PI conversion occurs prior to and independently from ER recruitment, and the two compartments appear to remain separate throughout the intracellular life of *L. pneumophila*.

Mechanistically, the PI conversion on the LCV possibly proceeds along several, mutually non-exclusive pathways: (i) the LCV might communicate and selectively retain PI-decorated vesicles, (ii) *L. pneumophila* might produce (Icm/Dot-secreted) effectors acting directly as PI interactors, phosphatases or kinases, and/or (iii) the pathogen might subvert host PI metabolizing enzymes (Figure 3). Indeed, using *D. discoideum* producing fluorescent PtdIns(3)*P* and PtdIns(4)*P* probes in tandem, we recently showed by high-resolution real-time confocal laser scanning microscopy that nascent LCVs continuously capture and accumulate PtdIns(4)*P*-positive vesicles derived from the *trans*-Golgi network (72). The sustained association of the PtdIns(4)*P*-positive vesicles, but not the LCV-vesicle interactions *per se*, require a functional T4SS. Thus, *L. pneumophila* exploits the cellular dynamics of vesicle-bound PtdIns(4)*P* for LCV formation. At different stages of infection *L. pneumophila* effectors might modulate the host PI pattern in different ways (73).

As outlined below in detail, *L. pneumophila* Icm/Dot-translocated effector proteins subvert PI lipids (i) by directly binding PIs (SidC, SidM, RidL, LtpM), (ii) by acting as bacterial PI phosphatases (SidF, SidP), PI kinases (LepB, LegA5), or phospholipases (VipD, PlcC, LpdA), or (iii) by recruiting eukaryotic PI phosphatases or kinases (RalF, SidM). Currently, no effector has been described, which directly modulates the activity of host PI-metabolizing enzyme. In general, *L. pneumophila* effectors determining the LCV PI pattern might act either *in cis* (on the LCV membrane) or *in trans* (in a distance from the LCV). In fact, a number of these effectors have been shown to act *in cis*, in agreement with their exceptional affinity for specific PI receptors (40, 74–76).

## Phosphoinositide Anchors for *L. Pneumophila* Effectors

**Figure 4:**
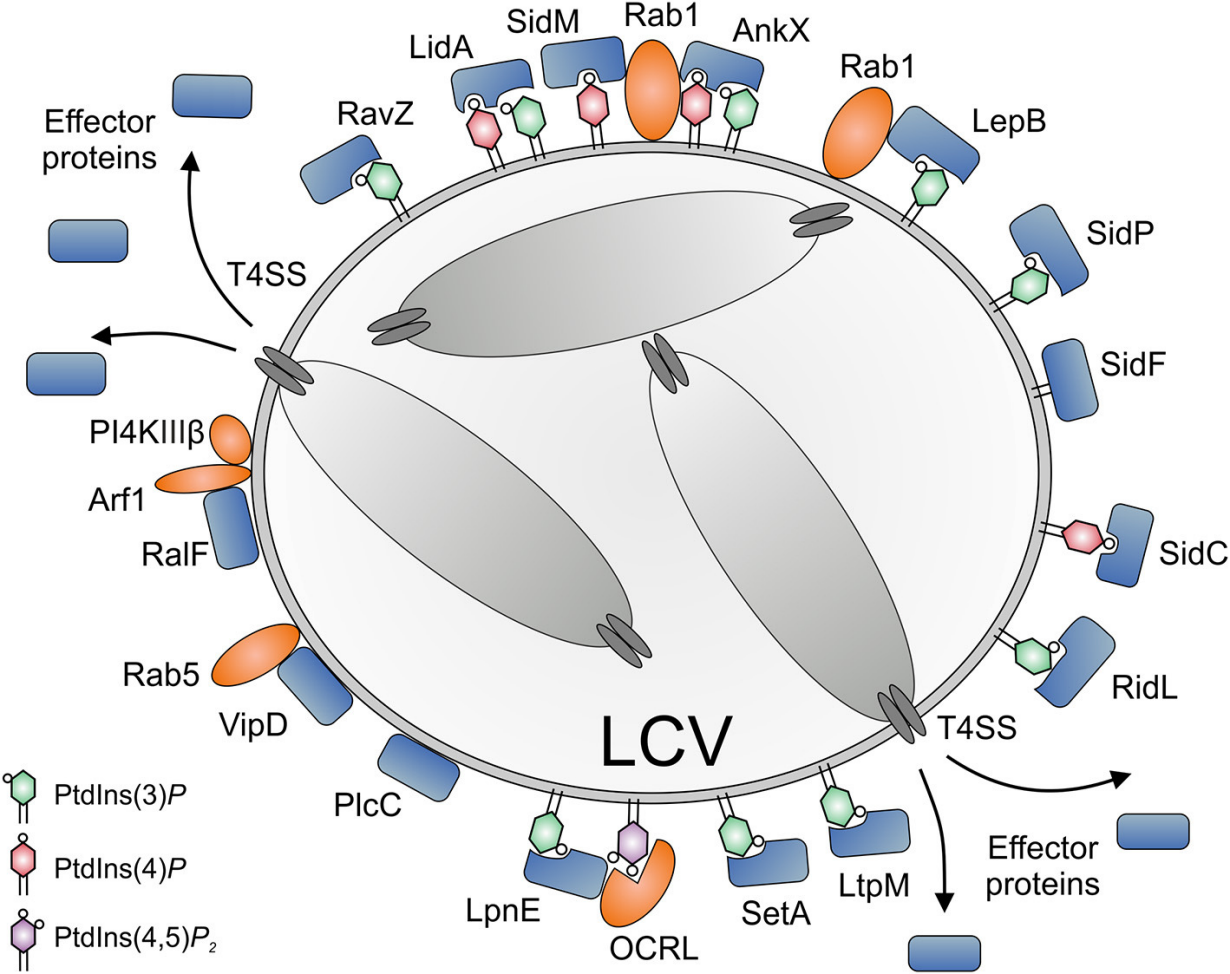
Subversion of host PI lipids by *L. pneumophila* effector proteins. *L. pneumophila* effector proteins translocated by the Icm/Dot T4SS subvert PI lipids on the *Legionella*-containing vacuole (LCV) (i) by directly binding PIs (SidC, SidM, AnkX, LidA, RidL, SetA, LtpM), (ii) by acting as bacterial PI phosphatases (SidF, SidP), PI kinases (LepB, LegA5), or phospholipases (VipD, PlcC, LpdA), or (iii) by recruiting eukaryotic PI phosphatases or kinases (RalF, SidM). PtdIns(4)*P* is bound by SidC (ubiquitin ligase) and SidM (Rab1 GEF/AMPylase). LidA and the Rab1 phosphocholinase AnkX bind PtdIns(3)*P* as well as PtdIns(4)*P*. PtdIns(3)*P* is bound by RidL (retromer inhibitor) and RavZ (Atg8/LC3 protease), as well as by SetA and LtpM (glycosyltransferases) and LepB (Rab1 GAP, PI 4-kinase). SidF and SidP are PI 3-phosphatases. VipD and PlcC function as a Rab5-activated phospholipase A_1_ or a Zn^2+^ metallophospholipase C, respectively. LpnE is secreted by an unknown mechanism and binds PtdIns(3)*P* as well as the host PI 5-phosphatase OCRL. The GEF RalF activates the small GTPase Arf1, which in turn recruits the host PI 4-kinase IIIβ (PI4KIIIβ). OCRL and PI4KIIIβ produce PtdIns(4)*P* from PtdIns(4,5)*P*_2_ or PtdIns, respectively.

*Legionella pneumophila* Icm/Dot substrates translocated to the cytoplasmic face of the LCV can bind to the pathogen vacuole as peripheral membrane protein [e.g., RalF; (77, 78)], as intrinsic membrane protein [e.g., MavN; (79, 80)], through host cell prenylation of a C-terminal CAAX motif [e.g., LegG1, AnkB, LpdA; (81–83)], or through PI lipids [e.g., SidC, SidM, RidL, LtpM; (44, 84, 85); Figure 4]. PI lipids bind a plethora of eukaryotic proteins through distinct domains (59), none of which was identified in *L. pneumophila* effector proteins. However, *L. pneumophila* produces a battery of effector proteins, which bind through novel domains to PtdIns(4)*P* (SidC, SdcA, SidM, Lpg1101, Lpg2603, AnkX, LidA) and/or PtdIns(3)*P* (LepB, RidL, SetA, LtpD, LtpM, RavD, RavZ, AnkX, LidA) (Table 1).

**Table 1:** *L. pneumophila* T4SS-translocated effectors targeting host PI lipids.

**Effector (*alias*)**	**Cellular target(s) and activity**	**References**
AnkX (LegA8/Lpg0695)	Rab1/Rab35 phosphocholinase, modulation of Rab1/Rab35 activity	(86–91)
LepB (Lpg2490)	Binding to PtdIns(3)*P*, Rab1 GAP, PI 4-kinase	(92–98)
LecE (Lpg2552)	Subversion of host phospholipid biosynthesis (DAG)	(99, 100)
LegA5 (Lpg2322)	Class III PI 3-kinase	(101)
Lem4 (Lpg1101)	Binding to PtdIns(4)*P*	(102)
Lem28 (Lpg2603)	Binding to PtdIns(4)*P*	(102)
LidA (Lpg0940)	Binding to PI lipids, protection of Rab1/Rab8 from GAPs	(103–107)
LpdA (Lpg1888)	Phospholipase D, hydrolysis of PG, PtdIns and PtdIns(3)*P*	(83, 99)
LppA (Lpg2819)	Inositol-P_6_ phosphatase (phytase), PI phosphatase activity *in vitro*	(108)
LtpD (Lpw3701)	Binding to PtdIns(3)*P* and (*myo*)-1-mono-phosphatase 1 (IMPA1)	(109)
LtpM (Lpp0356)	PtdIns(3)*P*-actived glycosyltranferase	(85)
PlcC (CegC1, Lpg0012)	Zn^2+^ metallophospholipase C, hydrolysis of PC, PG and PI	(110, 111)
RalF (Lpg1950)	Arf1/Arf6 GEF	(22, 77, 78, 112, 113)
RavD (Lpg0160)	Binding to PtdIns(3)*P*	(114)
RavZ	Binding to PtdIns(3)*P*, cysteine protease inhibiting autophagy	(48–50)
RidL (Ceg28/Lpg2311)	Binding to PtdIns(3)*P* and Vps29, inhibition of retrograde trafficking	(43–46)
SdcA (Lpg2510)	Binding to PtdIns(4)*P*, E3 ubiquitin ligase (mono-ubiquitination of Rab1), recruitment of ER to LCV	(51, 115–119)
SetA (Lpg1978)	Binding to PtdIns(3)*P*, UDP-glucosyltransferase, modification of histone H3.1 and H4 *in vitro*	(110, 120)
SidC (Lpg2511; Llo3098)	Binding to PtdIns(4)*P*, E3 ubiquitin ligase (mono-ubiquitination of Rab1), recruitment of ER to LCV	(51, 71, 75, 115–119, 121)
SidF (Lpg2584)	PI 3-phosphatase, hydrolysis of PI(3,4)*P*_2_ and PI(3,4,5)*P*_3_ *in vitro*	(122, 123)
SidM (DrrA/Lpg2464)	Binding to PtdIns(4)*P*, Rab1 GEF/AMPylase, modulation of Rab1/Rab35 activity	(38–40, 74, 84, 90, 92, 93, 102, 124–128)
SidP (Lpg0130)	PI 3-phosphatase, hydrolysis of PtdIns(3)*P* and PtdIns(3,5)*P*_2_ *in vitro*	(129)
VipD (Lpg2831)	Rab5-activated phospholipase A_1_, hydrolysis of PE, PC and PtdIns(3)*P*	(130–135)

*AMP, adenosine monophosphate; DAG, diacylglycerol; ER, endoplasmic reticulum; GAP, GTPase-activating protein; GEF, guanine nucleotide exchange factor; LCV, Legionella-containing vacuole; PC, phosphatidylcholine; PE, phosphatidylethanolamine; PG, phosphatidylglycerol; PI, phosphoinositide*.

The *L. pneumophila* Icm/Dot substrate SidC and its paralogue SdcA localize to the LCV membrane (115) and almost exclusively bind to PtdIns(4)*P* [(51); Figure 4 and Table 1]. The 105 kDa effector proteins harbor a unique 20 kDa C-terminal domain termed P4C [PtdIns(4)*P*-binding domain of SidC], which does not show similarity to any eukaryotic PI-binding motif and was used as a PtdIns(4)*P* probe in eukaryotic cells (116, 136). SidC and the P4C domain are conserved in *Legionella longbeachae*, where the 111 kDa effector represents the major PtdIns(4)*P* binding protein (75). The SidC orthologs of *L. pneumophila* and *L. longbeachae* bind PtdIns(4)*P* with a low dissociation constant (K_d_) of ca. 240 or 70 nM, respectively. The crystal structure of SidC revealed a unique PtdIns(4)*P*-binding domain essential for targeting the effector to the pathogen vacuole (137).

LCVs harboring an *L. pneumophila* Δ*sidC*-*sdcA* mutant strain recruit the ER slower and to a smaller extent; yet, the formation of the spatially separated PtdIns(4)*P*-positive limiting LCV membrane is not affected (28, 51, 65, 116). The interaction with the ER is catalyzed by a 70 kDa N-terminal fragment of SidC (116). The crystal structure of the N-terminal fragment revealed a novel fold (117, 121), comprising a catalytic Cys-His-Asp triad, which is essential for SidC to promote the polyubiquitination of protein substrates on the LCV (118). Indeed, SidC and SdcA act as E3 ubiquitin ligases, which show a broad and non-overlapping specificity for ubiquitin-conjugating E2 enzymes (118, 119). Hence, the *L. pneumophila* effector SidC links and subverts two different eukaryotic pathways, phosphoinositide and ubiquitination signaling.

In *L. pneumophila*-infected phagocytes, SidC decorates the LCV selectively, uniformly and in copious amounts (51, 116). We exploited this feature to isolate LCVs from homogenates of infected host cells by establishing a two-step procedure comprising immuno-affinity enrichment with an anti-SidC antibody, followed by Histodenz density gradient centrifugation (138, 139). Using this protocol, intact LCVs were isolated from *D. discoideum* amoeba (28, 140), murine RAW 264.7 macrophage-like cells (24, 27) and bone marrow-derived primary macrophages (141). The isolated LCVs were utilized for biochemical fusion experiments (28) and proteomics analysis (24, 27, 140, 141), which identified small GTPases and their effectors (Rab family, Rap1, Ran, RanBP1), large GTPases, components of the endosomal and late secretory trafficking pathways, as well as protein or lipid kinases and phosphatases. LCV localization of some of these proteins was confirmed by fluorescence microscopy using *D. discoideum* strains producing the corresponding GFP-fusion proteins (24, 26–28, 140, 142).

The Icm/Dot substrate SidM (*alias* DrrA) localizes to the LCV membrane early during *L. pneumophila* infection (92) and is the major PtdIns(4)*P*-binding protein, as it was exclusively identified as such in a non-biased pulldown approach [(84); Figure 4 and Table 1]. In lysates of *L. pneumophila* Δ*sidM*, no other PI-binding protein (not even SidC) was identified. The 73 kDa effector protein harbors the 12 kDa C-terminal domain P4M [PtdIns(4)*P*-binding domain of SidM], which does not show similarity to any eukaryotic PI-binding motif or the P4C domain of SidC, but is shared with two other effectors, Lpg1101 (*alias* Lem4) and Lpg2603 (*alias* Lem28) [(102); Table 1]. The P4M domain has been ectopically produced and used as a PtdIns(4)*P* probe in eukaryotic cells (143) and *Drosophila* photoreceptor cells (144). The crystal structure of SidM and biochemical analysis revealed a unique PtdIns(4)*P*-binding domain and a very high binding affinity (K_d_ = 4–18 nM) (40, 74).

SidM, i.e., its central domain, exerts GEF activity toward Rab1-GDI complexes, thus leading to GTP loading and Rab1 activation on LCV membranes (38, 39, 92, 124–127). Moreover, the N-terminal domain of SidM catalyzes the covalent attachment of AMP to Rab1, a reaction termed AMPylation (128), which renders Rab1(GTP) inaccessible to GAPs and causes the constitutive activation of the small GTPase on LCVs (93). The AMPylation reaction is reversible, and the *L. pneumophila* effector protein SidD can remove the AMP residue from Rab1 by a deAMPylation reaction (145–147). The removal of the covalent modification allows the GAP LepB to inactivate Rab1 (92, 94). Through activation of Rab1, SidM catalyzes the non-canonical pairing of plasma membrane t-SNARE syntaxin proteins (present on the LCV membrane) with the ER-localized v-SNARE protein Sec22b (148, 149). Thus, the SidM-catalyzed activation of Rab1 seems to promote the tethering and fusion of the LCV with ER-derived vesicles, which has been described many years ago (150, 151). In summary, the *L. pneumophila* effector SidM links and subverts two different eukaryotic pathways, phosphoinositide and small GTPase signaling.

The Icm/Dot substrate LidA supports SidM-dependent recruitment of Rab1 to LCVs (39) and preferentially binds to PtdIns(3)*P* or with lower affinity to PtdIns(4)*P* [(84, 103); Figure 4 and Table 1]. The 83 kDa effector targets Rab1 and several other host Rab GTPases (152, 153) and binds with high affinity to the GDP- and GTP-bound as well as the AMPylated form of Rab1, thus stabilizing the active conformation of the GTPase and preventing inactivation by GAPs (39, 104, 105).

The Icm/Dot substrate AnkX localizes to LCVs and binds with apparently similar affinity to PtdIns(3)*P* and PtdIns(4)*P* [(154); Figure 4 and Table 1]. AnkX covalently attaches a phosphocholine moiety to GDP-bound Rab1 and Rab35 in a process termed phosphocholination, which stabilizes inactive Rab1 at the LCV membrane (86, 87, 155). The CDP-choline-dependent activity of AnkX is reversed by the Icm/Dot-secreted effector Lem3, which dephosphocholinates Rab1 (88, 155).

The Icm/Dot substrate RidL specifically binds PtdIns(3)*P* and localizes to the LCV, juxtaposed to where the polar Icm/Dot T4SS connects to the pathogen vacuole membrane [(44); Figure 4 and Table 1]. RidL binds the Vps29 subunit of the retromer coat complex, inhibits retrograde trafficking and thereby promotes intracellular bacterial replication (19, 20). Structural studies revealed that a hydrophobic β-hairpin in the N-terminal domain of RidL interacts with Vps29, thus displacing the Rab7 GAP TBC1D5 [a regulator of retrograde trafficking; (43, 45, 46)].

The Icm/Dot substrate RavZ targets autophagosomes and binds PtdIns(3)*P* on high-curvature membranes trough a C-terminal domain [(49); Figure 4 and Table 1]. RavZ inhibits autophagy by deconjugating Atg8/LC3 from phosphatidylethanolamine (PE) (48). In contrast to the eukaryotic deconjugating factor Atg4, the cysteine protease RavZ irreversible decouples Atg8 from PE by hydrolyzing the amide bond between the C-terminal glycine and an adjacent aromatic amino acid in Atg8.

The Icm/Dot substrates SetA (110, 120) and LtpM (85) localize to LCVs and endosomes through C-terminal PtdIns(3)*P*-binding domains (Figure 4 and Table 1). The N-terminal domains of these effectors show similarities with glycosyl transferases, and indeed, the purified enzymes were found to exhibit glycohydrolase and glycosyltransferase activity *in vitro*, using UDP-glucose as a sugar donor. Intriguingly, PtdIns(3)*P* activates the glycosyltransferase activity of LtpM (85).

The Icm/Dot substrates LtpD (109) and RavD (114) also localize to the LCV through C-terminal PtdIns(3)*P*-binding domains (Table 1). LtpD might bind to the inositol monophosphatase IMPA1, which has indeed been detected on isolated LCVs (140). LpnE is a 41 kDa *L. pneumophila* virulence factor that binds to PtdIns(3)*P* and the eukaryotic PI 5-phosphatase OCRL (see below) [(156); Figure 4 and Table 1]. The Sel1 repeat-containing LpnE is secreted independently of the Icm/Dot T4SS or the Lsp T2SS and promotes uptake of *L. pneumophila* by phagocytes and intracellular replication (157, 158). Finally, a recent bioinformatics-based screen identified three novel PtdIns(3)*P*-binding domains, which are present in at least 14 known Icm/Dot substrates, including LepB and RavZ (95).

## *L. Pneumophila* Phosphoinositide Phosphatases, Kinases, and Phospholipases

*Legionella pneumophila* produces Icm/Dot-translocated effector proteins, which directly modify PI lipids by acting as PI phosphatases, PI kinases or phospholipases (Figure 4). The Icm/Dot substrate SidF localizes to the LCV at early time points of infection (2 h) [(122, 123); Figure 4 and Table 1]. The crystal structure of the N-terminal catalytic domain in complex with its substrate PtdIns(3,4)*P*_2_ revealed a positively charged groove in the catalytic center, similar to other PI phosphatases harboring the “CX_5_R” motif (123). The 102 kDa effector SidF harbors two predicted C-terminal transmembrane motifs, which anchor the protein to the LCV membrane. SidF specifically hydrolyses *in vitro* PtdIns(3,4)*P*_2_ and PtdIns(3,4,5)*P*_3_ typically occurring on early phagosomes, and it likely contributes to the production of PtdIns(4)*P* on LCVs, since vacuoles harboring *L. pneumophila* Δ*sidF* accumulate lower amounts of the PtdIns(4)*P*-binding effector SidC. Yet, the Δ*sidF* mutant strain is not impaired for intracellular growth.

The Icm/Dot substrate SidP acts as a PI 3-phosphatase *in vitro* and converts PtdIns(3,5)*P*_2_ to PtdIns(5)*P* as well as PtdIns(3)*P* to PtdIns (Figure 4 and Table 1). However, its PI-phosphatase activity was not assessed in *L. pneumophila*-infected cells, and a Δ*sidP* mutant strain is not impaired for intracellular growth (129). The crystal structure of SidP from *L. longbeachae* revealed three distinct domains: a large N-terminal catalytic domain, an appendage domain inserted into the catalytic domain, and a C-terminal α-helical domain. Based largely on biochemical studies, SidF and SidP were postulated to produce PtdIns(4)*P* and hydrolyze PtdIns(3)*P* on LCVs, thus contributing to the PI conversion on the pathogen vacuole.

The Icm/Dot substrate LepB is a Rab1 GAP (see above), but also shows PI 4-kinase activity specific for PtdIns(3)*P* [(96); Figure 4 and Table 1]. The effector might contribute to the production of PtdIns(4)*P* on LCVs, since pathogen vacuoles harboring *L. pneumophila* Δ*lepB* accumulate lower amounts of the PtdIns(4)*P*-binding effector SidC. LepB was proposed to convert PtdIns(3)*P* on LCVs into PtdIns(3,4)*P*, which could be hydrolyzed by SidF to yield PtdIns(4)*P* (96). Interestingly, the Icm/Dot substrate LegA5 (159), a membrane-associated effector toxic for yeast (110, 160), was recently found to be a wortmannin-insensitive, class III-like PI 3-kinase [(101); Table 1]. In fact, LegA5 might be a PI 3-kinase producing PtdIns(3)*P* on LCVs as a substrate for the PI 4-kinase LepB.

The Icm/Dot substrate LppA is another example of a CX_5_R motif PI phosphatase hydrolyzing *in vitro* PtdIns(3,4)*P*_2_, PtdIns(4,5)*P*_2_, and PtdIns(3,4,5)*P*_3_ to yield PtdIns(4)*P* [(108); Table 1]. While LppA appeared like an ideal candidate to produce PtdIns(4)*P* on LCVs, live-cell microscopy using GFP-P4C as a PtdIns(4)*P* probe indicated that LppA does not affect the LCV PI pattern. Instead, LppA is a T4SS-translocated hexakisphosphate inositol phosphatase (phytase), which degrades the micronutrient chelator phytate (indeed produced by amoebae), and thereby promotes the intracellular growth of *L. pneumophila*. Given that the *L. pneumophila* genome encodes more than 400 proteins with the CX_5_R (PI) phosphatase signature (123), other (PI) phosphatases are likely produced by the pathogen.

The Icm/Dot substrates VipD, PlcC, and LpdA are lipases, which possess broad range activity against phospholipids including mono-phosphorylated PIs (Figure 4 and Table 1). VipD was identified as an Icm/Dot substrate that impairs membrane trafficking in yeast (130, 131). The effector hydrolyzes PE as well as phosphatidylcholine (PC) (132) and, intriguingly, binds Rab5 as well as Rab22 and acts as a Rab5-activated phospholipase A_1_ (133–135). Accordingly, VipD removes PtdIns(3)*P* from endosomal membranes and thus might promote the evasion of the endocytic pathway by LCVs (133, 134). Analogously, the Icm/Dot substrate PlcC (*alias* CegC1) is a metallophospholipase C, which hydrolyzes a broad spectrum of lipids including PC, phosphatidylglycerol (PG), and PtdIns (111). The effector can degrade PtdIns(3)*P* and likely destabilizes target membranes. Finally, the Icm/Dot substrate LpdA is a phospholipase D that binds to membranes through C-terminal prenylation and hydrolyzes PG, PtdIns and PtdIns(3)*P* as well as PtdIns(4)*P* yielding phosphatidic acid (PA) (83). While LpdA does not seem to affect the cellular PI pattern, the phospholipase triggers Golgi fragmentation.

## Subversion of Host Phosphoinositide Kinases and Phosphatases by *L. Pneumophila*

In addition to directly modulating PI lipids, *L. pneumophila* effectors also subvert the host cell PI pattern indirectly by targeting eukaryotic PI phosphatases and kinases (Figure 4). The PtdIns(3)*P*-binding virulence factor LpnE binds mammalian OCRL (Oculocerebrorenal syndrome of Lowe) and its *Dictyostelium* homolog Dd5P4 (*D. discoideum* 5-phosphatase 4) via their N-terminal domains (156). The interaction of LpnE with OCRL was recently confirmed by size exclusion chromatography and supported by the crystal structure of the bacterial protein (161). OCRL and Dd5P4 are PI 5-phosphatases, which hydrolyse PtdIns(4,5)*P*_2_ and PtdIns(3,4,5)*P*_3_ to yield PtdIns(4)*P* and PtdIns(3,4)*P*_2_, respectively (162, 163). Dd5P4 is likely catalytically active on LCVs and increases the PtdIns(4)*P* available for binding by effectors such as SidC or SidM (156). Consequently, LpnE might increase the concentration of PtdIns(4)*P* on LCVs by recruiting OCRL/Dd5P4, and thereby promote PI conversion. *L. pneumophila* grows more efficiently in *D. discoideum* lacking Dd5P4, and thus, the pleiotropic PI 5-phosphatase restricts intracellular bacterial growth. Mechanistic details of this process are not known, but Dd5P4 modulates the recruitment of calnexin, Rab1 and retromer components to LCVs, which might account for growth restriction (156, 164).

The Icm/Dot substrates RalF and SidM possibly contribute indirectly to the modulation of the LCV PI pattern through the recruitment and activation of small host GTPases. RalF is an Arf1 GEF and activates the small GTPase on the LCV [(22, 112); Figure 4 and Table 1]. RalF harbors a C-terminal globular “capping” domain, which regulates GEF activity by auto-inhibition (77). Activated Arf1 recruits PI 4-kinase IIIβ (PI4KIIIβ) to the *trans* Golgi network (165), and hence, RalF might indirectly increase the PtdIns(4)*P* concentration on LCVs. Indeed, the depletion by RNA interference of PI4KIIIβ, but not PI4KIIIα or PI4KIIα decreases the amount of the PtdIns(4)*P*-binding effector SidC on LCVs, suggesting that in absence of PI4KIIIβ the level of PtdIns(4)*P* is reduced (84). Analogously, SidM recruits and activates Rab1 on LCVs (see above). Activated Rab1 (166) as well as Arf1 (167) recruit OCRL to endosomal membranes. Accordingly, SidM might not only bind to PtdIns(4)*P*, but also indirectly contribute to an increase of this PI on LCV membranes.

The Icm/Dot substrates LpdA and LecE localize to LCVs and might also indirectly modulate the LCV PI pattern by promoting DAG biosynthesis [(99); Table 1]. LpdA is a phospholipase D, which hydrolyzes PC to yield PA (see above). LecE enhances the activity of the eukaryotic PA phosphatase Pah1, which dephosphorylates PA yielding DAG. The second messenger DAG recruits protein kinase D (PKD) and its activator protein kinase C (PKC) to membranes. Activated PKD then interacts with PI4KIIIβ, thereby possibly also contributing to an increase in PtdIns(4)*P* on LCVs (99).

## Conclusions and Outlook

*Legionella pneumophila* replicates intracellularly in phagocytes within an LCV, a complex compartment tightly associated with the ER. The nascent LCV undergoes a PI conversion from PtdIns(3)*P* to PtdIns(4)*P*, and thereby is rerouted from the bactericidal endocytic to the replication-permissive secretory pathway. To modulate the PI pattern in infected cells, *L. pneumophila* (i) recruits PI-decorated vesicles, (ii) produces effectors acting as PI interactors, phosphatases, kinases or phospholipases, or (iii) subverts host PI-metabolizing enzymes. To this end, at least 21 T4SS-translocated effector proteins have been shown to target the host PI metabolism (Table 1). Intriguingly, a number of these effectors harbor 2–3 different functional domains and link PI signaling to other pivotal cellular pathways, e.g., SidC (PI interactor, ubiquitin ligase), SidM (PI interactor, Rab1 GEF, Rab1 AMPylase), LepB (PI interactor, PI 4-kinase, Rab1 GAP), SetA and LtpM (PI interactor, glycosyltransferase), and VipD (Rab5 interactor, phospholipase). LCV formation and the contribution of PI lipids to this process are incompletely understood. Among the more than 300 T4SS-translocated effector proteins of *L. pneumophila* only about 50 have been thoroughly investigated. Future studies will focus on the structural, molecular and cellular characterization of novel effectors implicated in host cell PI pattern subversion, as well as on the spatiotemporal regulation of effector translocation and function.

## Author Contributions

ALS and HH wrote the manuscript.

### Conflict of Interest

The authors declare that the research was conducted in the absence of any commercial or financial relationships that could be construed as a potential conflict of interest.

## Abbreviations

AMPylase: adenylyltransferase
DAG: diacylglycerol
Icm/Dot: intracellular multiplication/defective organelle trafficking
GAP: GTPase activating protein
GDI: guanine nucleotide dissociation inhibitor
GEF: guanine nucleotide exchange factor
LCV: *Legionella*-containing vacuole
OCRL: oculocerebrorenal syndrome of Lowe
PI: phosphoinositide
PI3/4/5K: PI 3-/4-/5-kinase
PtdIns: phosphatidylinositol
T4SS: type IV secretion system.
